# Enhanced exploration in reinforcement learning using graph neural network based intrinsic reward mechanism

**DOI:** 10.1038/s41598-025-23769-3

**Published:** 2025-11-14

**Authors:** J. Arun Pandian, Ramkumar Thirunavukarasu, Rajganesh Nagarajan

**Affiliations:** 1 School of Computer Science Engineering and Information Systems, Vellore Institute of Technology, Vellore, India; 2 Sri Venkateswara College of Engineering, Sriperumbudur, India

**Keywords:** Reinforcement learning, Exploration–exploitation trade-off, Graph neural networks, Intrinsic reward, State space exploration, Mathematics and computing, Computational science, Computer science, Information technology, Scientific data, Software, Statistics

## Abstract

We propose a Graph Neural Network-based Intrinsic Reward Learning (GNN-IRL) framework to address the exploration–exploitation trade-off in Reinforcement Learning (RL). This approach leverages the structural modeling capabilities of Graph Neural Networks (GNNs) to represent the transitions and relationships between states in the environment. Intrinsic rewards are computed based on centrality measures and inverse degree analysis within the state graph, enabling the agent to identify and explore novel or under-visited states. The effectiveness of GNN-IRL was validated in four benchmark environments with discrete action spaces: CartPole-v1, MountainCar-v0, Taxi-v3, and LunarLander-v3. Continuous state variables were discretized to construct state graphs, which facilitates the implementation of GNN-IRL but may limit scalability to very high-dimensional continuous spaces. The experimental results show that GNN-IRL outperforms state-of-the-art extrinsic and intrinsic exploration strategies in terms of convergence rate, cumulative reward, exploration efficiency, and state coverage. These findings demonstrate that GNN-IRL effectively balances exploration and exploitation, thereby improving sample efficiency and accelerating policy learning in discretized RL domains.

## Introduction

Reinforcement Learning (RL) is a branch of Machine Learning (ML) that provides effective solutions for sequential decision-making and control problems where autonomous learning is essential^1^. RL algorithms have demonstrated promising results in addressing challenges involving stochastic behavior and sample inefficiency^2^. In RL, the agent interacts with a dynamic environment and optimizes its actions based on the feedback received in the form of rewards^3^. The agent continuously learns by exploring various states, performing actions, and observing the outcomes. The primary goal of an RL algorithm is to train the agent to derive an optimal action-selection policy through interaction with the environment^4^. RL has been successfully applied to diverse domains, such as object detection^5^, resource optimization^6^, personalized recommendation^7^, manufacturing^8^, finance and trading^9^, healthcare^10^, and logistics^11^.

One of the major challenges in RL is the well-known exploration–exploitation dilemma^12^. Balancing exploration (discovering new states) and exploitation (using known information to maximize rewards) is key to improving the learning performance of an agent^13^. Without an effective exploration strategy, the agent may become trapped in local minima and fail to discover optimal policies. Efficient exploration involves understanding the environment, collecting informative experiences, ranking the transitions, and adapting policies to maximize the cumulative rewards.

Classical extrinsic exploration techniques, such as Epsilon-Greedy, Boltzmann Exploration, Thompson Sampling, and Upper Confidence Bound (UCB), are widely used in both tabular and deep RL settings^14^. These methods typically rely on probabilistic selection strategies but lack awareness of the structural relationships among states. Consequently, they perform poorly in unfamiliar or sparse-reward environments.

Intrinsic exploration techniques have been introduced to address these limitations. It was inspired by intrinsic motivation in psychology, where behavior is driven by internal rewards rather than external stimuli^15^. In RL, intrinsic rewards act as internal signals that encourage exploration, particularly in environments with sparse or deceptive reward structures^16^. These rewards are typically based on measures such as curiosity, surprise, or state novelty. The most common intrinsic techniques are curiosity-driven, count-based, bipartite preference, and entropy-based techniques. Intrinsic methods can lead to suboptimal policies if internal reward signals diverge from task objectives. Furthermore, these methods may struggle with high-dimensional or complex state-action spaces, leading to inefficient exploration or redundant behaviour.

There is a growing need for sophisticated exploration strategies that enable agents to navigate complex environments efficiently, avoid revisiting uninformative states, and prioritize structurally important regions^17^. In this context, Graph Neural Networks (GNNs) offer a powerful mechanism for modeling state transitions and guiding exploration. GNNs are a class of artificial neural networks designed to operate on graph-structured data^18^. They have demonstrated superior performance in domains where the relationships among entities can be naturally represented as graphs. In RL, GNNs can help agents identify important states and model the environment effectively. Structuring the state space as a graph enables better generalization, faster learning, and informed exploration of previously unseen states^19^.

This study proposes a GNN-based Intrinsic Reward Learning (GNN-IRL) framework to enhance the exploration efficiency of RL agents. The framework models the environment as a dynamic graph in which nodes represent states and edges represent transitions. The agent uses GNNs to learn graph embeddings that capture the structural and semantic information of the environment. Intrinsic rewards are computed based on graph metrics, such as centrality and inverse degree, encouraging the agent to explore both influential and underrepresented states. The proposed GNN-IRL is integrated with a Deep Q-Network (DQN), enabling effective learning in environments with discrete action spaces.

The key contributions of this study are summarized as follows:

- A novel intrinsic reward learning method, GNN-IRL, was proposed to enhance the decision-making policy of RL algorithms.
- The framework leverages GNNs to compute intrinsic rewards from the graph representations of the environment.
- Dynamic graph models state-action transitions, and the intrinsic reward is derived from node centrality and inverse degree.
- The proposed method was integrated into the DQN model and evaluated in discrete action space environments.
- Experimental validation was conducted on benchmark discrete action space environments to demonstrate improvements in the cumulative reward, convergence, and exploration quality.
- The proposed GNN-IRL method outperformed several state-of-the-art extrinsic and intrinsic exploration strategies across key performance metrics.

The remainder of this paper is organized as follows: “Related research” reviews existing exploration techniques and their limitations. “Graph neural network-based intrinsic reward learning” presents the design of the GNN-IRL framework and the experimental setup. “Results and discussion” provides a comparative evaluation of the baseline methods. Finally, “Conclusions and future works” concludes the study and discusses the future research directions.

## Related research

In Reinforcement Learning (RL), exploration refers to the process by which an agent discovers new states and actions to maximize its cumulative reward^20^. Effective exploration is essential, particularly when an agent faces sparse rewards or complex state-action spaces that include large local optima. A simple exploration strategy is epsilon-greedy, where a time-decaying parameter ε controls the trade-off between exploration and exploitation. Although this approach is simple and performs reasonably well in structured environments such as Atari games^21^. The random selection of ε values often leads to inefficient learning in large or continuous state-action spaces. In contrast, Boltzmann exploration introduces a temperature parameter (τ) to balance the probability distribution of action selection. In^22^, this method was used by robotic agents to navigate unknown environments by optimizing the trade-off between finding new paths and reusing familiar paths. As an alternative, Upper Confidence Bound (UCB) techniques prioritize actions with uncertain outcomes by computing confidence bounds on the expected rewards. In^23^, UCB was applied to explore states and actions with high uncertainty to improve the Q-value estimation. In contrast, Thompson Sampling (TS) samples from the posterior distribution of the Q-values and uses the same Q-function throughout an episode. This leads to a deeper exploration over longer episodes, especially in sparse reward scenarios^24^. Most RL agents that follow extrinsic reward mechanisms face the Noisy-TV problem^25^. The agent may become trapped in cycles of exploration that yield no useful rewards or encounter environments with delayed rewards that prevent learning meaningful behavior. These situations can cause agents to converge to suboptimal policies.

Intrinsic reward mechanisms have been introduced to address the challenges faced by traditional extrinsic reward mechanisms^26^. These mechanisms assign additional internal rewards based on novelty or surprise, encouraging agents to explore, even when extrinsic rewards are sparse or delayed. This self-motivation helps agents discover informative parts of the state space and promotes better generalization of the model. Count-based methods estimate novelty by tracking the frequencies of state visitation. Although effective, this approach is difficult to scale in larger discrete or continuous state spaces. To alleviate this, researchers have applied count-based techniques to reduced-dimensional feature spaces^27^. However, this still requires significant memory and careful feature design^28^. Entropy-based exploration aims to maximize the uncertainty of state transitions by promoting actions that result in unpredictable outcomes. In^29^, entropy was used to guide exploration in sparse reward environments, such as Montezuma’s Revenge^30^, resulting in a strong performance.

Curiosity-driven exploration is another widely adopted intrinsic reward method, in which agents are rewarded based on the surprisingness or novelty of their experiences. These methods are often categorized into state-novelty and state-action novelty estimations^26,31^. In^31^, the authors applied state-novelty for anomaly detection using intrinsic unsupervised rewards. In^26^, a forward dynamics model was used to compute rewards based on the prediction error of the next state, which guided exploration in partially observable environments. A prominent curiosity-driven technique is Random Network Distillation (RND)^32^, which assigns intrinsic rewards based on the prediction error between a fixed, randomly initialized target network and a trainable predictor network. When the agent visits novel states, the prediction error is high, resulting in stronger intrinsic rewards. As familiar states are revisited, the predictor improves and the intrinsic reward decreases, naturally encouraging exploration. Although RND avoids explicit state counting and offers scalability advantages, it requires high memory and computational resources in high-dimensional or continuous state spaces. Furthermore, because RND ignores environmental transition dynamics, it may perform poorly in stochastic or noisy environments.

The bipartite policy for reward shaping (BiPaRS) is a more recent method that employs two policies: one for the main task and another for generating adaptive shaping rewards^33^. This architecture allows the agent to dynamically adjust the reward structure based on its progress. BiPaRS demonstrated superior performance in environments with sparse or misleading rewards. However, its effectiveness depends on well-coordinated learning between the two policies, which can increase the training complexity and instability.

Despite these advancements, most intrinsic exploration methods suffer from high memory usage, difficulty in feature representation, inefficiency in very sparse environments, and poor scalability in high-dimensional settings. Table 1 summarizes the types and key limitations of prominent exploration techniques in reinforcement learning.

**Table 1:** Summary of existing exploration techniques.

Technique	Type	Limitations
Bipartite preference (e.g., BiPaRS)	Intrinsic	Requires coordination between two policies; lacks direct modeling of state structure.
Boltzmann	Extrinsic	Struggles in sparse reward settings; limited deep exploration capabilities.
Count-based	Intrinsic	High memory usage; difficult to apply in continuous state spaces.
Curiosity-driven (e.g., RND)	Intrinsic	Ignores transition dynamics; high computational demand in large environments
Entropy-based	Intrinsic	Sensitive to noise; difficult to tune entropy-based parameters.
Epsilon-Greedy	Extrinsic	Inefficient in large state-action spaces; depends heavily on ε decay schedule.
Thompson sampling	Extrinsic	Relies on accurate posterior sampling; may underperform in complex dynamics.
UCB	Extrinsic	Assumes known confidence bounds; scales poorly in high-dimensional spaces.

Graph Neural Networks (GNNs) are neural architectures designed to process graph-structured data by modeling the dependencies between entities^34^. GNNs have gained popularity in applications such as combinatorial optimization and relational reasoning^35^. Q-learning^36^ is a fundamental RL algorithm that estimates the optimal policy using a tabular method and Bellman updates. However, it becomes inefficient in high-dimensional state-action spaces. Deep Q-Networks (DQN)^37^ were introduced to overcome the limitation. It approximates the Q-function using neural networks and improves the scalability and generalization. In this study, we propose a GNN-based intrinsic reward mechanism that leverages graph structural features, such as centrality and inverse degree, to incentivize exploration. The Q-value function is modeled using a GNN within the DQN framework. At each time step, the agent receives graph-based state observations and learns policies for action selection. The proposed GNN-IRL framework is described in the following section.

### Graph neural network-based intrinsic reward learning

**Fig. 1:**
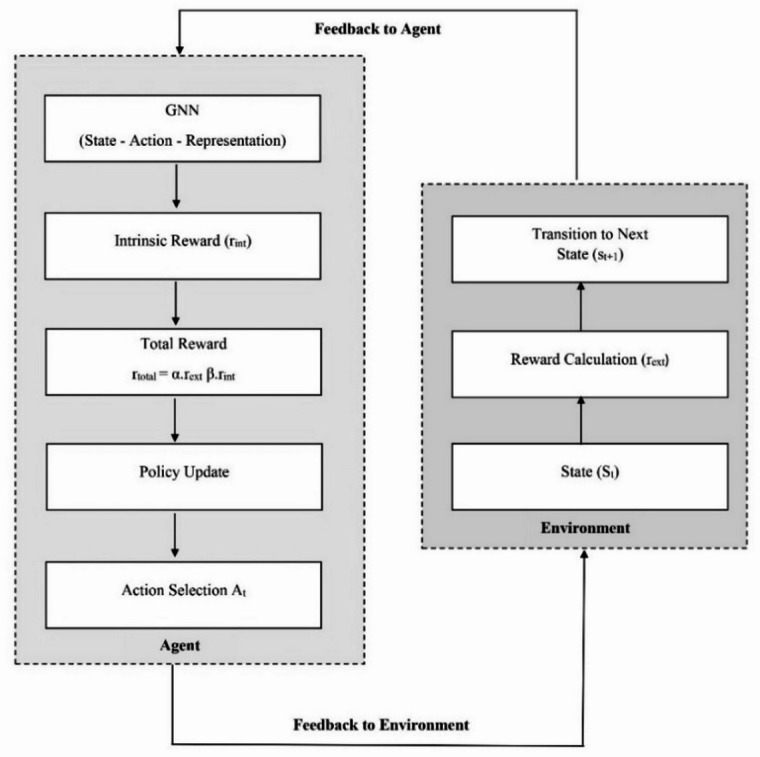
Layered architecture of the proposed Framework.

The exploration process in RL techniques discovers new states in the environment by selecting different actions from the action space. Exploring suitable states in the environment is crucial for improving the decision-making policy of RL techniques. The proposed exploration technique uses a GNN to enhance the exploration process in RL techniques. The proposed framework calculates the intrinsic reward using a dynamic graph of state-action transitions. The nodes and edges of the graph represent the states and transitions, respectively. The proposed exploration technique is called Graph Neural Network-based Intrinsic Reward Learning (GNN-IRL). It combines the strengths of GNNs with the Intrinsic reward mechanisms to control exploration in RL techniques. Figure 1 shows the interaction between the RL agent, GNN model, and intrinsic reward mechanism.

The subsequent subsections discuss how the states, actions, and transitions are represented by GNNs, the computation of intrinsic rewards using the properties of GNNs, and the interaction between the RL agent and the environment.

### Graph neural network model

**Fig. 2:**
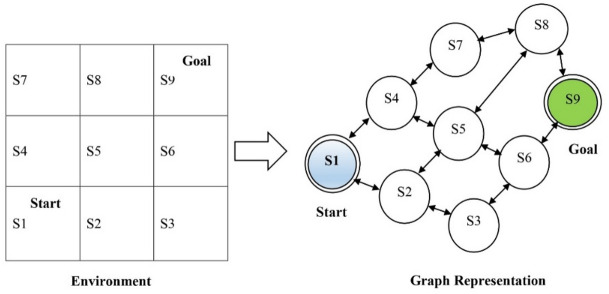
Graph representation of state transition.

GNNs are a special type of deep neural network that can represent data in graph structures. However, traditional deep neural networks represent data using vectors. The nodes in the graphs characterize the entities, and the edges signify the relationships or transitions between the entities. GNNs are highly suitable for learning the complex relationships between entities in relational data. GNNs were used in this study to enhance the learning performance of the RL algorithms by visualizing the structure and dynamics of the environment for agents. In the proposed GNN-IRL, the states in the environment are denoted by the nodes of the GNN, and the action or state transitions are represented by their edges. Figure 2 illustrates the graphical representation of the transition between the states in the grid world environment.

The transitions between states (weights or edges) were computed using the probability of transitioning from state s_i_ to s_j_ after selecting action a. The edge weight *w*_*ij*_ is defined as Eq. 1.

1 $$\:{w}_{ij}=P({s}_{j}\mid\:{s}_{i},a)$$

Where *P* is the transition probability between states s_i_ and s_j_ while performing action a.

The edge weights in the constructed state-transition graph were computed based on the estimated transition probabilities. For each observed transition from state s to a subsequent state s′ under action a, an empirical transition table was maintained, and an estimate P(s′|s, a) was made using normalized transition counts accumulated over episodes. This allows the edge (s, s′) in the graph to be weighted proportionally to the likelihood of observing a transition. This enables the agent to effectively model the structural dynamics of the environment. Although the exact transition probabilities are not known a priori, this approach incrementally approximates them through repeated environmental interactions, making it well-suited for stochastic but fully observable settings with discrete state spaces. In cases of high environmental stochasticity, the transition graph naturally encodes the uncertainty via distributed edge weights. This modeling strategy facilitates the reliable computation of graph-theoretic measures used for intrinsic-reward generation.

Graph embeddings were used to capture the structural and semantic information of the environments. Graph embeddings are vector representations of graph components, such as nodes and edges. Graph embeddings map high-dimensional graph data into a continuous low-dimensional vector space. The vector representation of states and transitions is useful in reinforcement learning for improving computational efficiency, particularly in large and complex environments. Each node in the graph characterizes the unique state of the environment in the RL. The edges represent actions or transitions between states. Node embedding describes the properties of individual states, including their relationships with neighboring states. The node embeddings iteratively learn about the nodes during the training process. The learning process of node embedding is controlled by Eq. 2.

2 $$\:{\text{h}}_{\text{j}}^{\left(\text{k}\right)}={\upsigma\:}\left({\text{W}}^{\left(\text{k}\right)}.\:\:{\sum\:}_{\text{i}\in\:\text{N}\left(\text{j}\right)}\frac{{\text{w}}_{\text{i}\text{j}}}{\sqrt{{\text{d}}_{\text{i}}{\text{d}}_{\text{j}}}}{\text{h}}_{\text{i}}^{(\text{k}-1)}+{\text{b}}^{\left(\text{k}\right)}\right)$$

Where $\:{\text{h}}_{\text{j}}^{\left(\text{k}\right)}$ is the embedding of the state j at the layer k. N(j) denotes the neighboring states of state j. The $\:{\text{W}}^{\left(\text{k}\right)}$ and $\:{\text{b}}^{\left(\text{k}\right)}$ are the learnable weight and bias are at layer k. The $\:{\upsigma\:}$ is a nonlinear activation function. The $\:{\text{d}}_{\text{i}}{\:\text{a}\text{n}\text{d}\:\text{d}}_{\text{j}}$ are the degrees of the nodes i and j.

Node embedding uses a Graph Convolutional Network (GCN) to encode graphs into vector data. The RL agent used the embedding of the state-action graph to compute the novelty of a transition between states by calculating the intrinsic rewards.

### Intrinsic reward mechanism

Intrinsic rewards were used in this study to motivate the RL agent to learn about the environment by exploring unvisited and minimally visited states. Intrinsic rewards ensure that the agent not only learns to maximize the rewards but also effectively explores the environment to discover new states. The Intrinsic rewards were calculated internally in the agent based on the properties of the state transition graph. The centrality and inverse degree of a state of the environment were used to compute the intrinsic rewards in this study.

The centrality of the state measures its importance in the state space of the environment. This study employed the centrality measure to calculate the intrinsic reward to explore the central or influential state, which is commonly connected with most of the states. It is used to support the agent in visiting most states in the environment. The calculation of state centrality is shown in Eq. 3.

3 $$\:\text{C}\text{e}\text{n}\text{t}\text{r}\text{a}\text{l}\text{i}\text{t}\text{y}\left(\text{s}\right)=\frac{\sum\:_{j\in\:N\left(s\right)}{w}_{ij}}{\sum\:_{i,j\in\:\:S}{w}_{ij}}$$

Where $\:{w}_{ij}$ is the edge weight between states i to j. This was calculated using Eq. 1.

The inverse degree of a state signifies the reciprocal of its degree. The degree of a state represents the number of directly connected adjacent states. The inverse degree gives higher importance to less connected states in the environment. This factor encourages exploration of the RL agent. Equation 4 is used to compute the inverse degree of the state.

4 $$\:InverseDegree\left(s\right)=\:\frac{1}{\left|N\left(s\right)\right|}$$

Where the $\:\left|N\left(s\right)\right|$ is the number of neighboring states connected to s.

The combination of the centrality and inverse degree of the state helps the agent strategically explore both dominant and underrepresented states. This ensures a more balanced and efficient exploration strategy for the RL agent. The calculation of Intrinsic reward $\:{r}_{int}$ is defined in Eq. 5.

5 $$\:{r}_{int}\left(s\right)=\alpha\:\cdot\:Centrality\left(s\right)+\beta\:\cdot\:InverseDegree\left(s\right)$$

where *α* and *β* are the scaling factors for controlling the influence of the centrality and inverse degree in the calculation of the intrinsic reward *r*_*int*_*(s)*. These scaling factors are important hyperparameters that control the contribution of centrality and inverse degree to the intrinsic reward calculation.

The Extrinsic rewards ($\:{r}_{ext}$) is obtained by the agent after selecting the action. The Extrinsic reward function was defined by an environment that evaluates the success of the action selected by the agent. The Extrinsic reward estimation was calculated using Eq. 6.

6 $$\:{r}_{ext}({s}_{i},a,{s}_{j})=f({s}_{i},a,{s}_{j})$$

where *s*_*i*_ and *s*_*j*_ represent the current and next states, respectively, and *a* denotes the action selected in state *s*_*i*_ at time t.

The total reward signal was calculated using the intrinsic and extrinsic rewards of the state-action pairs. The total reward optimizes the policy of the RL agent for the action selection. The total reward signal was computed using Eq. 7.

7 $$\:{r}_{total}=x\cdot\:{r}_{ext}+y\cdot\:{r}_{int}\text{}$$

where x and y are the scaling factors for controlling the influence of intrinsic and extrinsic rewards in the total reward calculation. These scaling factors control the influence of intrinsic and extrinsic rewards in the total reward estimation.

The convergence was tested using the stopping criteria for the proposed GNN-IRL framework, which were defined to ensure efficient and meaningful termination of the training process of the RL agent. The training process stops when the RL agent meets at least one of the following three conditions.

i. Maximum total reward: the total reward, which is the sum of the extrinsic and intrinsic rewards, reaches its maximum value.

8 $$\:{r}_{total}\:\ge\:\:{r}_{max}-\mu\:$$

Where the $\:\mu\:$ is a stabilization value and $\:{r}_{max}$ is the maximum possible reward that can be obtained.

ii. Intrinsic reward convergence: the intrinsic reward reaches zero or a predefined threshold.

9 $$\:{r}_{int}\:\le\:\:\delta\:$$

where *δ* represents the minimum possible threshold for the intrinsic reward. It is either zero or nearly so.

iii. Novel states unchanged: the number of novel states $\:{S}_{novel}\left(t\right)$ remains unchanged over consecutive episodes. This signifies that the agent has completely explored the state space. It shows that no new states are discovered by the agent for exploration.

10 $$\:{S}_{novel}\left(t\right)={S}_{novel}(t-1)=\cdots\:={S}_{novel}(t-M)$$

Where M is a predefined patience parameter, and t is the current training episode.

The pseudocode algorithm of the proposed GNN-IRL approach uses the GNN and Intrinsic reward addition to the existing strategy, which is presented in Algorithm 1.

**Algorithm 1:**
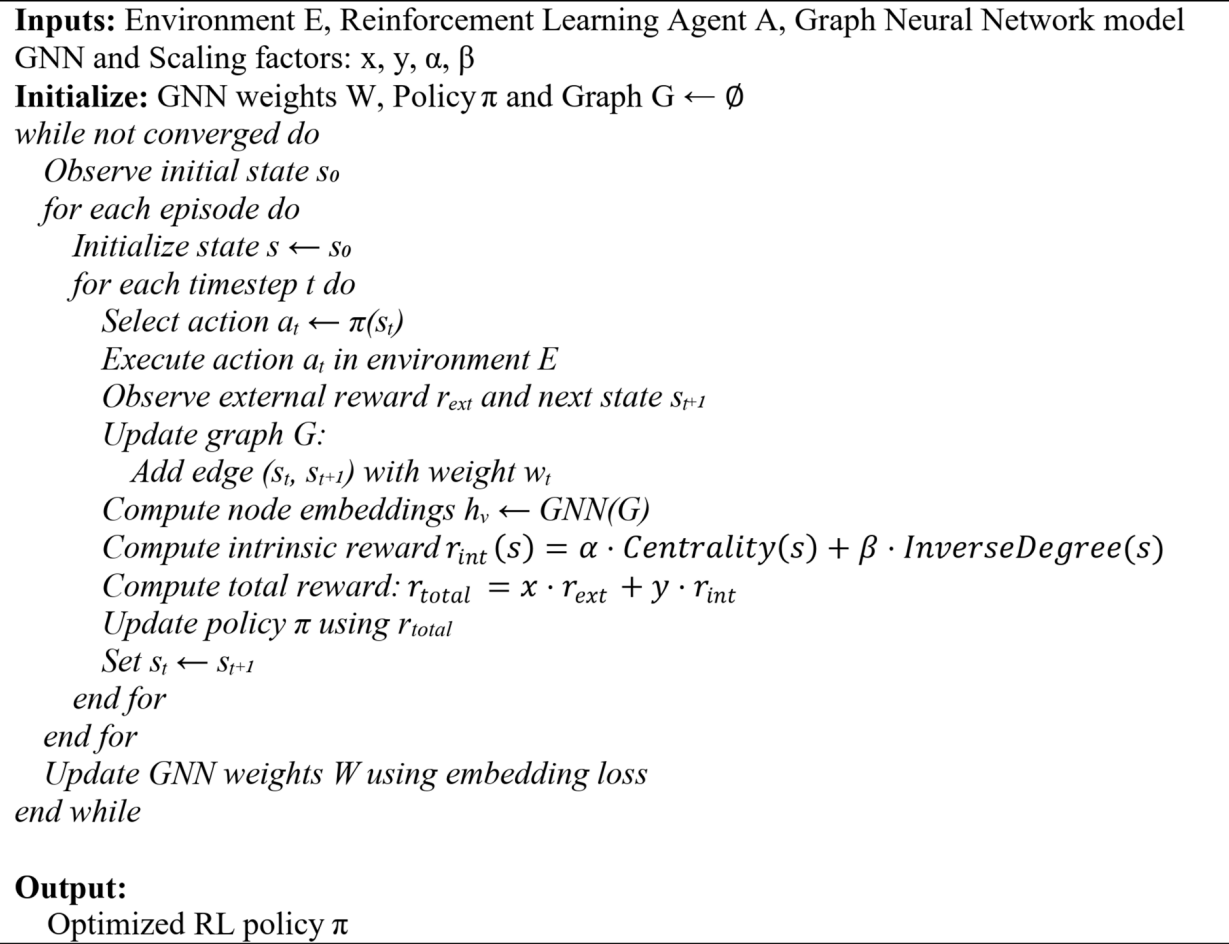
GNN-IRL for exploration.

### Experimental setup and hyperparameter tuning

The proposed GNN-IRL was combined with the DQN algorithm to learn about discrete action space environments, such as CartPole-v1, MountainCar-v0, Taxi-v3, and LunarLander-v3. Popular classical control environments were used to implement and validate the proposed GNN-IRL technique. Hyperparameters play a crucial role in improving the performance of machine-learning algorithms. The selection of suitable hyperparameter values is an important process for obtaining optimal performance from learning techniques. This study used a random search technique to tune the hyperparameter value selection. First, the hyperparameter values of the GNN for the CartPole-v1 environment were optimized using a random search technique. The optimized hyperparameter values of the GNN model for the CartPole-v1 environment are listed in Table 2.

**Table 2:** Hyperparameter values of GNN.

Hyperparameter	Value	Description
Number of Layers	3	Number of GNN layers for message passing.
Hidden Layer Size	64	Number of units in each hidden layer of the GNN.
Activation Function	ReLU	Non-linear activation function for GNN layers.
Dropout Rate	0.3	Dropout rate for regularization during training.
Learning Rate (GNN)	0.003	Learning rate for GNN-specific optimization.
Aggregation Function	Mean	Method to aggregate messages from neighboring nodes.
Node Embedding Dimension	32	Size of the learned node embeddings.
Message Passing Steps	2	Number of iterations for message passing.
Optimizer	Adam	Optimization algorithm for updating weights.

The Intrinsic reward mechanism is the most important in the proposed GNN-IRL approach to assist the RL agent in choosing the most appropriate action from the action space. Some hyperparameter values need to be optimized in the intrinsic reward calculation to enhance the performance of the RL agent. The tuned hyperparameter values of the intrinsic reward mechanism for the CartPole-v1 environment are listed in Table 3.

**Table 3:** Hyperparameter values of intrinsic reward mechanism.

Hyperparameter	Value	Description
Inverse Degree Factor	0.2	Scaling factor for inverse degree centrality contribution.
Intrinsic Reward Decay	0.96	Decay rate to reduce intrinsic reward over episodes.
Novel State Threshold	5	The threshold to determine if a state is considered novel.

The proposed GNN-IRL exploration technique was implemented on the DQN model to validate its performance and efficiency in different environments. Hence, the hyperparameters and their optimized values of the DQN technique for the CartPole-v1 environment were tuned, as shown in Table 4.

**Table 4:** Hyperparameter values of DQN Model.

Hyperparameter	Value	Description
Learning Rate	0.0005	Learning rate for the RL agent.
Discount Factor (γ)	0.99	Weight given to future rewards.
Max Episodes	500	Total number of episodes for training.
Max Steps per Episode	200	Maximum Number of steps allowed in each episode.
Replay Buffer Size	10,000	Size of the buffer for experience replay.
Batch Size	32	Number of samples per training step.
Optimizer	Adam	Optimization algorithm for updating weights.

**Fig. 3:**
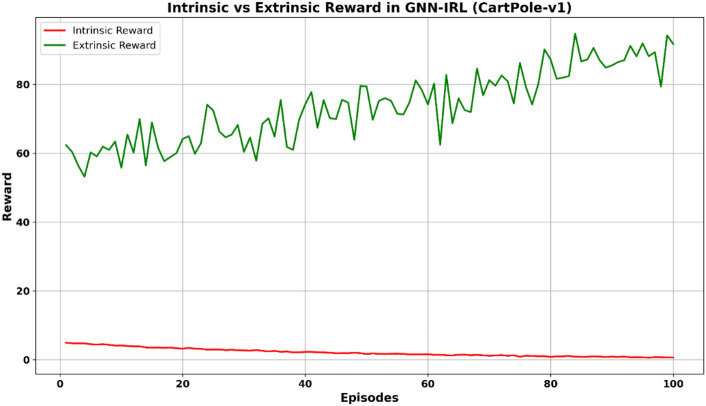
Training performance of the GNN-IRL.

The episode-wise rewards, intrinsic rewards, and novel states explored by the agent were used to validate the efficiency and reliability of the proposed GNN-IRL for control problems. The training performance of the proposed GNN-IRL exploration technique in CartPole-v1 is shown in Fig. 3.

The continuous improvement in episode-wise rewards and the corresponding decline in intrinsic reward values observed in the CartPole-v1 environment demonstrate the consistency and stability of the proposed GNN-IRL framework. Furthermore, the GNN-IRL-enhanced DQN model, equipped with optimized hyperparameters, was evaluated on additional benchmark environments, including MountainCar-v0, Taxi-v3, and LunarLander-v3. These experiments aimed to maximize the cumulative rewards and enhance the decision-making capabilities of the agent. The quantitative and qualitative results, along with the limitations of the proposed GNN-IRL exploration approach, are discussed in the subsequent sections. In addition, the framework was compared with several widely adopted and recent exploration techniques using standard performance evaluation metrics.

## Results and discussion

The proposed GNN-IRL exploration approach was implemented using the DQN algorithm to learn and generate a policy for action selection. Standard control environments, such as cartpole, mountain car, taxi, and lunar lander, were used to analyze the performance of the proposed GNN-IRL and baseline exploration strategies. The baseline exploration strategies used in this study were BiPaRS, Boltzmann exploration, count-based exploration, epsilon-greedy exploration, RND, entropy-based exploration, Thompson sampling exploration, and UCB exploration.

### Experimental results

**Fig. 4:**
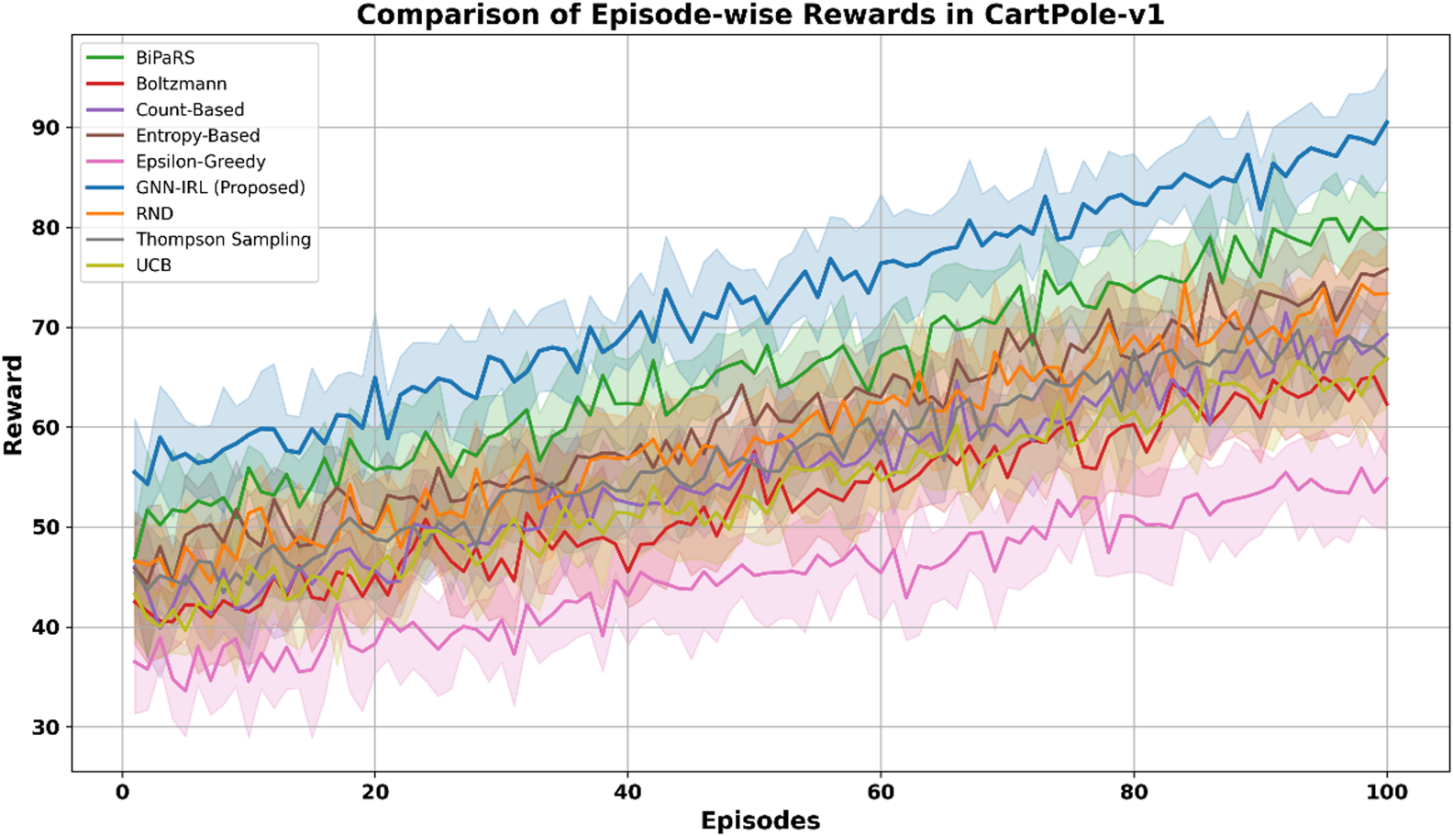
Comparison of episode-wise rewards in CartPole-v1.

The episode-wise reward trajectories of the proposed GNN-IRL framework were compared with those of several state-of-the-art exploration strategies, including RND, BiPaRS, Boltzmann, Count-Based, Entropy-Based, Epsilon-Greedy, Thompson Sampling, and UCB, across four discrete-action benchmark environments: CartPole-v1, MountainCar-v0, Taxi-v3, and LunarLander-v3. The average episode-wise rewards over 10 independent runs were used to ensure a fair comparison and to mitigate the stochastic variance in individual trials. Both the mean reward and standard deviation were computed for each episode across the runs. A state discretization process was applied to facilitate the implementation of the GNN-IRL and enable graph construction from continuous or hybrid state spaces. In the CartPole-v1 environment, each dimension of the continuous state vector was discretized using 10 uniform bins per dimension, resulting in 10⁴ = 10,000 unique discrete states. Figure 4 illustrates the learning progression in CartPole-v1, with shaded areas representing ± 1 standard deviation, covering approximately 68% of the observed values to indicate variability. The GNN-IRL consistently outperformed the baseline methods, achieving faster convergence and higher final rewards, whereas strategies such as Epsilon-Greedy and Count-Based exhibited slower and noisier learning owing to less structured exploration.

**Fig. 5:**
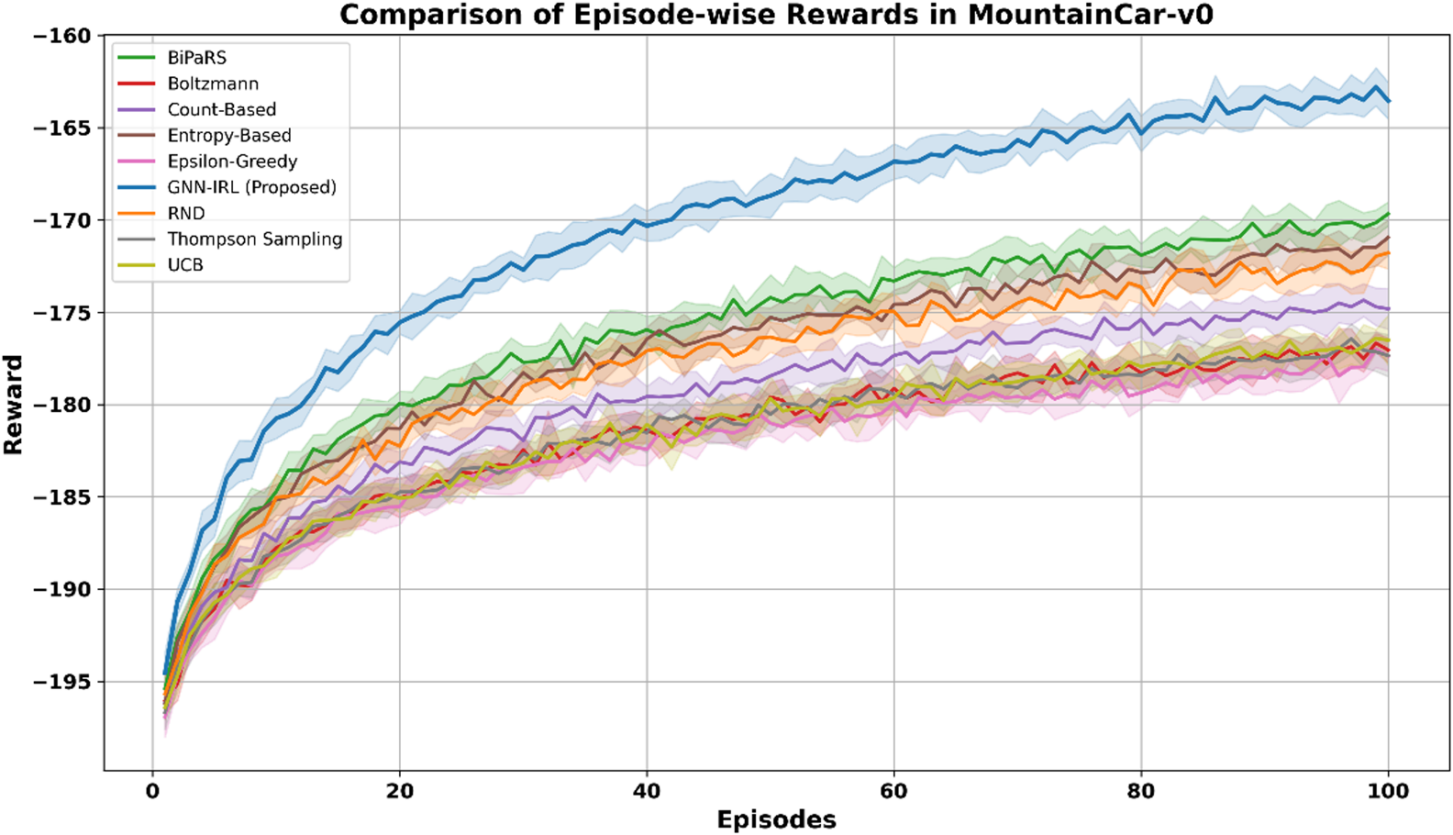
Comparison of episode-wise rewards in MountainCar-v0.

MountainCar-v0 uses 20 bins per dimension to capture finer motion and velocity resolution, producing 400 unique discrete states. These discrete states form the nodes in the environment graph, with edges representing state transitions owing to agent actions. Figure 5 presents the results for the MountainCar-v0 environment, which features sparse and delayed reward. GNN-IRL demonstrated superior performance by quickly discovering the optimal trajectory to reach the goal state. Its structured exploration, driven by graph-based intrinsic rewards, allows for more effective navigation of the state space compared to random or count-based methods, which often struggle with convergence in this domain.

**Fig. 6:**
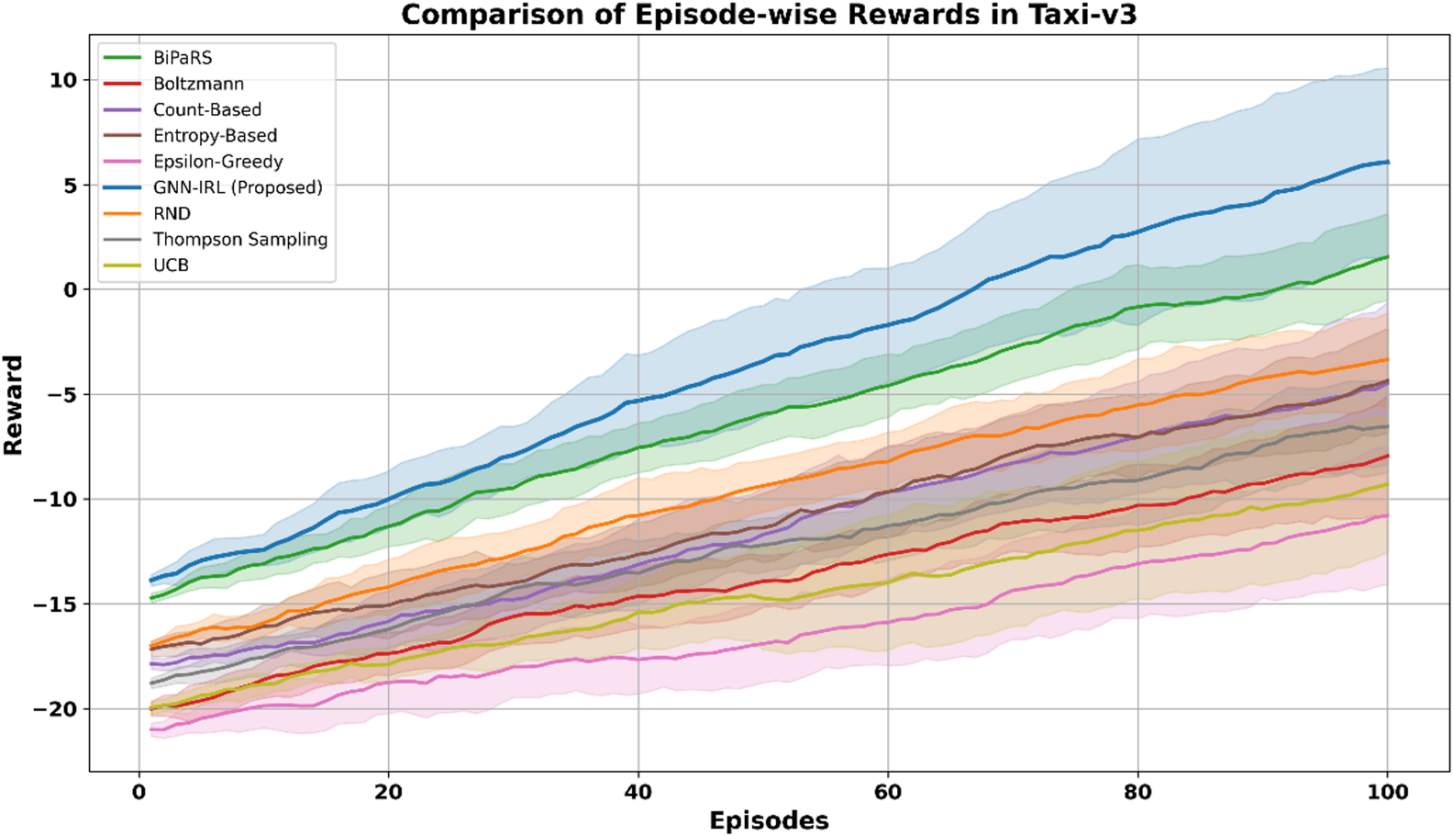
Comparison of episode-wise rewards in Taxi-v3.

The Taxi-v3 environment is inherently discrete and grid-based, consisting of 500 distinct states defined by the position of the agent, passenger location, and the destination. Therefore, no additional discretization was required. The structured transitions of the environment allowed for straightforward graph construction, facilitating efficient GNN operations on the experience of the agent. The Taxi-v3 environment, characterized by a discrete grid layout with dynamic task-dependent transitions, such as varying passenger pickup and drop-off locations, presents a structured yet challenging exploration setting. As shown in Fig. 6, the proposed GNN-IRL framework achieved faster reward accumulation and a more stable learning curve than the baseline strategies. This improvement highlights the ability of the model to exploit topological information from the state transition graph of the environment, thereby enabling efficient navigation and consistent policy learning in discrete, goal-conditioned environments.

**Fig. 7:**
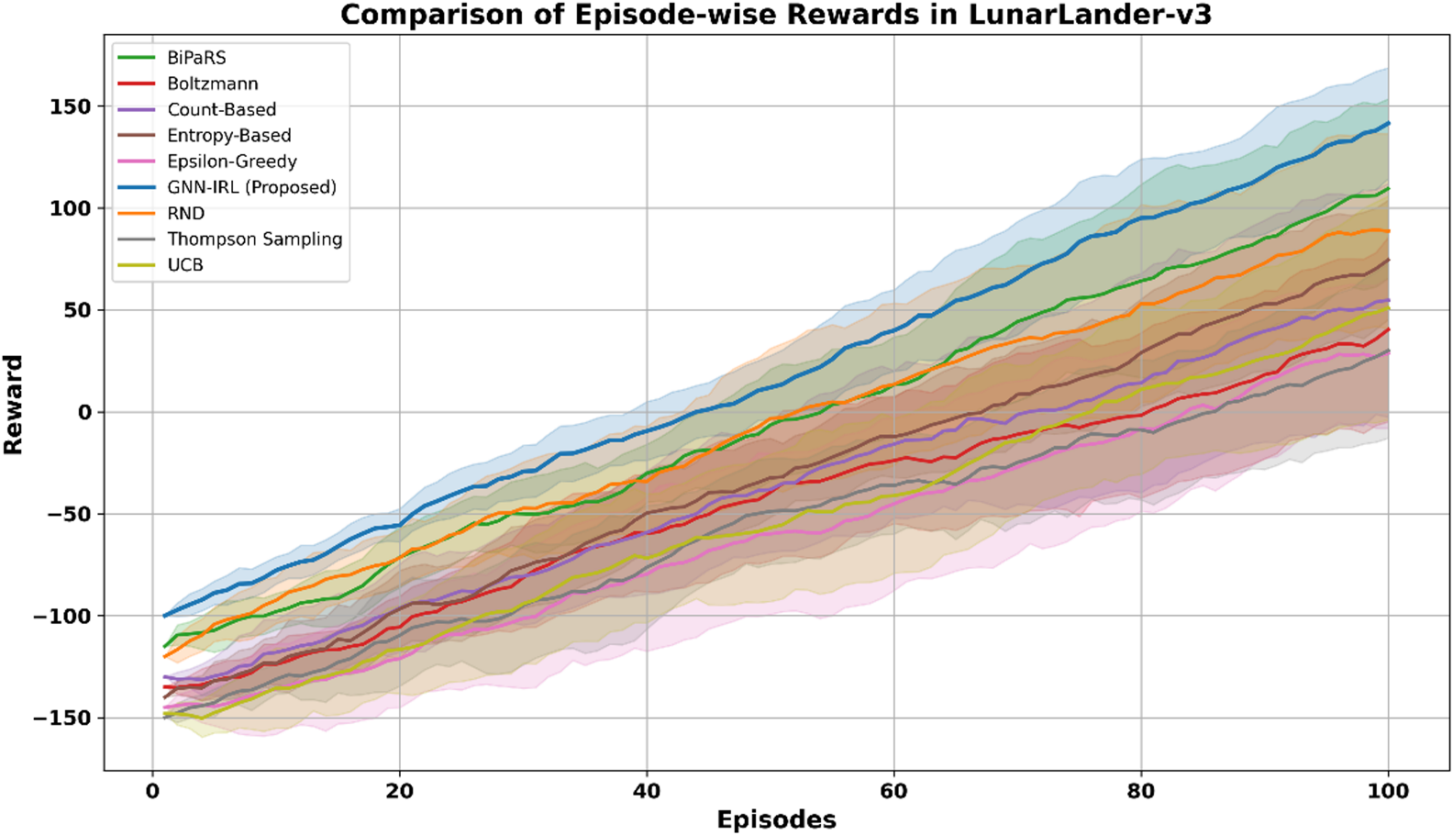
Comparison of episode-wise rewards in LunarLander-v3.

In LunarLander-v3, although the environment has continuous state components, a discretization scheme was applied by dividing each state dimension (e.g., position, velocity, angle, and leg contacts) into five bins depending on sensitivity to reward dynamics. This produced 5,000 distinct discrete states after removing unreachable combinations, enabling the GNN to compute intrinsic rewards based on the structural properties of the discretized environment graph. Figure 7 shows the performance in the LunarLander-v3 environment, which is characterized by stochastic dynamics and moderately sparse rewards. The GNN-IRL approach maintained a smooth and steady learning curve and attained higher cumulative rewards than all baseline techniques. Its ability to consistently identify and prioritize novel and informative states results in efficient policy development, even under uncertain transitions.

Overall, these results, averaged across multiple training runs, confirm the stability, scalability, and robustness of the GNN-IRL in diverse discrete-action environments. Structured discretization enables the effective construction of environment graphs, which, combined with intrinsic motivation based on graph metrics, allows GNN-IRL to significantly improve exploration efficiency and reward optimization across tasks.

### Ablation study

An ablation study was conducted to evaluate the individual contributions of the intrinsic reward components to the proposed GNN-IRL framework. Specifically, this study examined three configurations: (i) centrality-based intrinsic rewards only, (ii) inverse degree–based rewards only, and (iii) a combined formulation using various α/β weight settings. The performance was measured using a cumulative reward metric. The cumulative reward measures the total reward accumulated over fixed episodes. The cumulative reward is computed using Eq. 11:

11 $$\:cumulative\:reward=\:\sum\:_{i=1}^{E}{r}_{ext}\left(i\right)$$

Where E is the total number of episodes. The cumulative reward ranges from -∞ to +∞. A higher cumulative reward denotes a greater performance of the RL agents.

As presented in Table 5, the combined approaches consistently outperformed individual components across all benchmark environments. In particular, the equal weighting configuration (α = 0.5, β = 0.5) achieved the highest cumulative reward, highlighting the complementary nature of centrality and inverse degree metrics in driving effective exploration.

**Table 5:** Ablation study of intrinsic reward components in GNN-IRL.

Configuration	α	β	CartPole-v1	MountainCar-v0	Taxi-v3	LunarLander-v3
Centrality only	1.0	0.0	1108	-123	-285	19
Inverse degree only	0.0	1.0	1122	-118	-175	26
Equal weights	0.5	0.5	1189	-98	-9	32
Centrality weighted higher	0.7	0.3	1157	-105	-85	13
Inverse degree weighted higher	0.3	0.7	1162	-101	-53	16

Table 6 presents an ablation study analyzing the impact of different weight configurations between intrinsic (x) and extrinsic (y) rewards in the GNN-IRL framework across the four benchmark environments. The results show that using only intrinsic or extrinsic rewards yields suboptimal performance. Equal contribution provides moderate improvements, whereas assigning a higher weight to intrinsic rewards (x = 0.7, y = 0.3) consistently leads to superior cumulative rewards in all environments. This highlights that placing more importance on intrinsic motivation enhances exploration and accelerates learning, especially in sparse or deceptive reward settings.

**Table 6:** Ablation study of scaling factors in GNN-IRL.

Configuration	x	y	CartPole-v1	MountainCar-v0	Taxi-v3	LunarLander-v3
Intrinsic only	1.0	0.0	1143	-107	-38	13
Extrinsic only	0.0	1.0	1105	-125	-43	02
Equal contribution	0.5	0.5	1153	-113	-15	26
Intrinsic weighted higher	0.7	0.3	1189	-98	-9	32
Extrinsic weighted higher	0.3	0.7	1170	-104	-27	21

The two ablation studies collectively demonstrate the effectiveness of both the structural components and reward balancing strategy within the GNN-IRL framework. Overall, the findings confirm that leveraging a hybrid reward mechanism grounded in structural graph features and appropriately balanced with extrinsic feedback greatly improves the learning efficiency and agent performance across diverse environments.

### Computational cost analysis

A comparative analysis of different discretization resolutions across benchmark environments highlights the inherent trade-off between the computational cost and cumulative reward. Table 7 presents the computational cost of GNN-IRL for various discretization configurations.

**Table 7:** Computational cost analysis of GNN-IRL under different discretization Configurations.

Environment	Discretization	#States	Cost (s/episode)	Cumulative reward
CartPole-v1	5 bins/dim	625	0.12	1050
	10 bins/dim	10,000	0.98	1189
	20 bins/dim	160,000	6.5	1195
MountainCar-v0	10 bins/dim	100	0.05	–140
	20 bins/dim	400	0.14	–98
	40 bins/dim	1600	0.45	–95
Taxi-v3	Discrete grid	500	0.01	–9
LunarLander-v3	3 bins/dim	500	0.25	–10
	5 bins/dim	5,000	1.80	32
	10 bins/dim	50,000	12.3	34

In CartPole-v1, increasing the number of bins from 5 to 10 significantly improved the performance, but further refinement to 20 bins yielded only marginal gains while causing a steep rise in the computational overhead. A similar trend was observed in MountainCar-v0, where the 20-bin configuration achieved the best balance, as finer discretization provided only slight improvements in the reward but at a substantial computational cost. For Taxi-v3, the environment is inherently discrete, eliminating the need for additional discretization while delivering strong results. In LunarLander-v3, increasing the number of bins from 3 to 5 markedly enhanced the cumulative rewards, but moving to 10 bins offered negligible additional benefits despite dramatically higher costs. Collectively, these findings confirm that the chosen discretization strategies—10 bins per dimension for CartPole-v1, 20 bins per dimension for MountainCar-v0, the natural grid for Taxi-v3, and five bins per dimension for LunarLander-v3—represent the optimal compromise, ensuring high rewards with manageable computational demands.

### Performance analysis

The performance of the exploration techniques was assessed using standard performance metrics, such as convergence rate, convergence time, exploration efficiency, and state coverage. The average performance metric values of ten independent runs for each method were used for the comparison. The convergence rate represents the inverse of the number of episodes the RL agent takes to reach the threshold reward ($\:{r}_{threshold})$. The convergence rate is calculated using Eq. 12.

12 $$\:\text{C}\text{o}\text{n}\text{v}\text{e}\text{r}\text{g}\text{e}\text{n}\text{c}\text{e}\:\text{R}\text{a}\text{t}\text{e}=\:\frac{1}{{E}_{\text{t}\text{h}\text{r}\text{e}\text{s}\text{h}\text{o}\text{l}\text{d}}}$$

Where $\:{E}_{\text{t}\text{h}\text{r}\text{e}\text{s}\text{h}\text{o}\text{l}\text{d}}$ is the episode taken for the agent to reach the $\:{r}_{threshold}$. The convergence rate ranged from 0 to 1. A higher convergence rate indicates better performance.

The convergence time represents the total time (T) taken to reach the $\:{r}_{threshold}$. Equation 13 is used to compute the convergence time of the exploration techniques.

13 $$\:Convergence\:Time\:\left(seconds\right)=\:T\left({r}_{threshold}\right)$$

The exploration efficiency indicates the number of unique states visited during the training of the RL agent. This refers to the percentage of episodes in which the agent successfully explores novel states. A higher exploration efficiency indicates that the RL agent explores more states during training. Equation 14 shows how the exploration efficiency is calculated.

14 $$\:\text{E}\text{x}\text{p}\text{l}\text{o}\text{r}\text{a}\text{t}\text{i}\text{o}\text{n}\:\text{e}\text{f}\text{f}\text{i}\text{c}\text{i}\text{e}\text{n}\text{c}\text{y}\:\left(\text{\%}\right)=\:\frac{\left|Unique\:States\:Explored\right|}{\left|Total\:States\:Visited\right|}\:\times\:100$$

Moreover, state coverage is the proportion of the environment explored during training. The state coverage of the RL agent was computed using Eq. 15:

15 $$\:\text{S}\text{t}\text{a}\text{t}\text{e}\:\text{c}\text{o}\text{v}\text{e}\text{r}\text{a}\text{g}\text{e}\:\left(\text{\%}\right)=\:\frac{\left|Unique\:States\:Explored\right|}{\left|Total\:States\:in\:Environment\right|}\:\times\:100$$

The comparison results of the proposed GNN-IRL and existing exploration techniques in the CartPole-v1 environment are presented in Table 8. The GNN-IRL framework demonstrated superior performance on all evaluation metrics. It achieved the highest convergence rate of 0.0089 and the lowest convergence time of 1344 s, indicating faster policy learning. Additionally, it obtained the highest cumulative reward of 1189 and state coverage of 76%, highlighting its effectiveness in balancing exploration and exploitation compared with the baseline methods.

**Table 8:** Performance comparison in CartPole-v1.

Technique	Convergence rate	Convergence time	Cumulative reward	Exploration efficiency	State coverage
BiPaRS	0.0061	1980	1134	0.61	0.69
Boltzmann	0.0037	3216	996	0.45	0.51
Count-based	0.0036	3312	1093	0.55	0.62
Entropy-based	0.0052	2292	1076	0.54	0.61
Epsilon-Greedy	0.0034	3504	917	0.42	0.48
GNN-IRL	0.0089	1344	1189	0.68	0.76
RND	0.0056	2172	1109	0.59	0.66
Thompson sampling	0.0043	2808	944	0.59	0.66
UCB	0.0037	3252	973	0.57	0.64

Table 9 presents the results for the MountainCar-v0 environment. With 400 discrete states and a convergence threshold of 50, the proposed GNN-IRL method again demonstrated superior performance, reaching the convergence threshold in only 1469 s. It achieved a higher cumulative reward of -98, along with the highest exploration efficiency and state coverage among all the compared techniques. These results confirm the effectiveness of GNN-IRL in navigating sparse-reward continuous state space environments through graph-based intrinsic motivation.

**Table 9:** Performance comparison in MountainCar-v0.

Technique	Convergence rate	Convergence time	Cumulative reward	Exploration efficiency	State coverage
BiPaRS	0.0061	2040	-114	81	85
Boltzmann	0.0039	3328	-155	68	72
Count-Based	0.0038	3432	-156	72	75
Entropy-Based	0.0056	2327	-134	76	79
Epsilon-Greedy	0.0036	3640	-161	65	70
GNN-IRL	0.0088	1469	-98	88	90
RND	0.0055	2360	-127	78	80
Thompson Sampling	0.0045	2886	-138	66	71
UCB	0.0039	3367	-147	67	73

Taxi-v3 is a grid-based environment with dynamic task-based transitions, which also benefits from the proposed approach. As shown in Table 10, the proposed GNN-IRL method achieved the highest cumulative reward of -9, the least convergence time of 1259 s, and the widest state coverage of 91%. These results highlight the ability of GNN-IRL to adaptively guide exploration in structured environments with sparse and delayed reward signals.

**Table 10:** Performance comparison in Taxi-v3.

Technique	Convergence rate	Convergence time	Cumulative reward	Exploration efficiency	State coverage
BiPaRS	0.0071	1635	-42	83	85
Boltzmann	0.0041	2980	-139	69	72
Count-based	0.004	3096	-122	71	74
Entropy-based	0.0058	2150	-85	76	79
Epsilon-Greedy	0.0038	3248	-158	66	70
GNN-IRL	0.0092	1259	-9	89	91
RND	0.0066	1782	-68	79	82
Thompson sampling	0.0049	2673	-128	68	71
UCB	0.0042	2928	-133	67	72

The GNN-IRL model achieved the best overall performance in the LunarLander-v3 environment, as presented in Table 11. It recorded the highest cumulative reward of 32 and led across all exploration metrics, including convergence rate, efficiency, and state coverage.

**Table 11:** Performance comparison in LunarLander-v3.

Technique	Convergence rate	Convergence time	Cumulative reward	Exploration efficiency (%)	State coverage (%)
BiPaRS	0.0072	1758	21	81	84
Boltzmann	0.0043	2704	-38	64	69
Count-Based	0.0053	2250	-13	73	76
Entropy-Based	0.0061	1984	-5	78	80
Epsilon-Greedy	0.0039	2921	-46	61	64
GNN-IRL	0.0091	1402	32	85	88
RND	0.0065	1896	4	79	81
Thompson Sampling	0.0047	2508	-26	68	72
UCB	0.0045	2620	-30	66	70

Table 12 also reports the standard deviations of the cumulative rewards from 10 independent trials. This demonstrates that GNN-IRL not only outperforms other techniques but also exhibits the highest consistency and stability across all environments.

**Table 12:** Standard deviation of cumulative rewards.

Technique	CartPole-v1	MountainCar-v0	Taxi-v3	LunarLander-v3
BiPaRS	47.2	4.3	15.5	11.7
Boltzmann	58.6	8.2	22.4	17.3
Count-based	53.3	7.9	20.1	15.7
Entropy-based	51.4	5.8	17	12.4
Epsilon-Greedy	60.2	9.7	24.6	18.9
GNN-IRL	35.6	2.5	9.3	6.8
RND	50.9	6.7	13.9	14.3
Thompson sampling	57.5	8.6	21.2	16.6
UCB	59.1	8.3	22	17.1

The standard deviation values presented above demonstrate the robustness and consistency of the GNN-IRL technique in different environments. The GNN-IRL consistently exhibited the lowest standard deviation in the cumulative reward in all four environments, indicating higher training stability and more predictable performance. These results further reinforce that GNN-IRL not only achieves superior average rewards but also maintains a reliable learning behavior over multiple training runs.

The experimental results show that the proposed GNN-IRL achieves a superior convergence rate, convergence time, cumulative reward, exploration efficiency, and state coverage compared with existing techniques in sparse-reward and high-dimensional environments. The proposed GNN-IRL uses graph centrality and inverse degree to calculate intrinsic rewards. This technique prioritizes novel states and balances exploration and exploitation during the training process. This technique provides advantages such as systematic and adaptive exploration, scalability to high-dimensional environments, and interpretable reward design for RL agents.

### Limitations of the proposed approach

Although the proposed GNN-IRL framework demonstrated superior performance across discrete-action environments, it has certain limitations. Its effectiveness depends on the quality of the constructed state-transition graph, and poorly structured or sparse graphs can reduce the informativeness of the intrinsic rewards. The computation of graph-based metrics at each step introduces additional overhead, which may affect the scalability of large or high-dimensional state spaces. The current formulation is designed for discrete or discretized states, limiting its direct applicability to continuous or hybrid environments, and maintaining the graph requires significant memory. Additionally, the performance can be sensitive to hyperparameters, such as discretization resolution and reward weighting. Despite these limitations, GNN-IRL provides a systematic and interpretable approach to exploration, with opportunities for future extensions to continuous actions and more efficient graph representation.

## Conclusions and future works

This study proposed a novel GNN-IRL framework to address the challenge of efficient exploration in RL. GNN-IRL leverages the representational capabilities of Graph Neural Networks to model relationships among states, enabling the agent to better understand the structural layout of the environment. Intrinsic rewards are computed using graph-theoretic metrics, such as centrality and inverse degree, which encourage the exploration of novel and informative regions of the state space. Experimental evaluations across discrete-action benchmark environments, including CartPole-v1, MountainCar-v0, Taxi-v3, and LunarLander-v3, showed that GNN-IRL consistently outperformed existing strategies, such as Boltzmann, Count-Based, Curiosity-Driven, Entropy-Based, Epsilon-Greedy, Thompson Sampling, and UCB, achieving higher cumulative rewards, faster convergence, improved exploration efficiency, and broader state coverage. The ability of GNN-IRL to identify structurally significant states through node representations enables more effective exploration policies and accelerated learning, particularly in sparse or deceptive reward environments. The dynamic adaptation of intrinsic rewards based on evolving graph structures further enhances agent performance in complex or delayed-reward scenarios. However, performance is influenced by the quality and granularity of the constructed state-transition graph, and the computational overhead of graph analysis may limit the scalability in very large or continuous state spaces. Future research directions include extending GNN-IRL to continuous control problems, high-dimensional observation spaces and multi-agent systems. Integrating transfer learning to improve cross-domain adaptability, implementing the approach in real-time physical systems such as robotics or autonomous navigation, and exploring hybrid reward shaping strategies that combine graph-based intrinsic motivation with learned reward priors could further enhance its practical applicability and performance in dynamic environments.

## Data Availability

The data generated and analyzed during the current study are available from the corresponding author on reasonable request.

## References

[1] Pan, J. & Wei, Y. A deep reinforcement learning-based scheduling framework for real-time workflows in the cloud environment. *Expert Syst. Appl.* **255**, 124845 (2024).

[2] Andersen, P. A., Goodwin, M. & Granmo, O. C. Towards safe and sustainable reinforcement learning for real-time strategy games. *Inf. Sci.* **679**, 120980 (2024).

[3] Shakya, A. K., Pillai, G. & Chakrabarty, S. Reinforcement learning algorithms: A brief survey. *Expert Syst. Appl.* **231**, 120495 (2023).

[4] David, J. et al. The use of reinforcement learning algorithms in object tracking: A systematic literature review. *Neurocomputing* **596**, 127954 (2024).

[5] Yao, H., Dong, P., Cheng, S. & Yu, J. Regional attention reinforcement learning for rapid object detection. *Comput. Electr. Eng.* **98**, 107747 (2022).

[6] Zhou, X., Yang, J., Li, Y., Li, S. & Su, Z. Deep reinforcement learning-based resource scheduling for energy optimization and load balancing in SDN-driven edge computing. *Comput. Commun.* **226**, 107925 (2024).

[7] Halder, S., Lim, K. H., Chan, J. & Zhang, X. A survey on personalized itinerary recommendation: from optimisation to deep learning. *Appl. Soft Comput.* **152**, 111200 (2024).

[8] Wang, L., Pan, Z. & Wang, J. A review of reinforcement learning based intelligent optimization for manufacturing scheduling. *Complex. Syst. Model. Simul.* **1**, 4, 257–270 (2021).

[9] Cheng, L. C. & Sun, J. S. Multi-agent-based deep reinforcement learning framework for multi-asset adaptive trading and portfolio management. *Neurocomputing* **594**, 127800 (2024).

[10] Zhong, C. et al. A new cloud-based method for composition of healthcare services using deep reinforcement learning and Kalman filtering. *Comput. Biol. Med.* **172**, 108152 (2024). 38452470 10.1016/j.compbiomed.2024.108152

[11] Aboutorab, H., Hussain, O. K., Saberi, M. & Hussain, F. K. A reinforcement learning-based framework for disruption risk identification in supply chains. *Future Generation Comput. Syst.* **126**, 110–122 (2022).

[12] Chen, Q., Zhang, Q. & Liu, Y. Balancing exploration and exploitation in episodic reinforcement learning. *Expert Syst. Appl.* **231**, 120801 (2023).

[13] Ladosz, P., Weng, L., Kim, M. & Oh, H. Exploration in deep reinforcement learning: A survey. *Inform. Fusion*. **85**, 1–22 (2022).

[14] Hao, J. et al. Exploration in deep reinforcement learning: from single-agent to multi-agent domain. *IEEE Trans. Neural Networks Learn. Syst.* **35**, 7, 8762–8782 (2023). 10.1109/TNNLS.2023.3236361 37021882

[15] Moran, M. & Gordon, G. Deep curious feature selection: A recurrent, intrinsic-reward reinforcement learning approach to feature selection. *IEEE Trans. Artif. Intell.* **5** (3), 1174–1184 (2024).

[16] Zhao, A. et al. Self-referencing agents for unsupervised reinforcement learning. *Neural Netw.* **188**, 107448 (2025). 40198945 10.1016/j.neunet.2025.107448

[17] Waikhom, L. & Patgiri, R. A survey of graph neural networks in various learning paradigms: methods, applications, and challenges. *Artif. Intell. Rev.* **56** (7), 6295–6364 (2023).

[18] Zhou, J. et al. Graph neural networks: A review of methods and applications. *AI open.* **1**, 57–81 (2020).

[19] Lu, H., Wang, L., Ma, X., Cheng, J. & Zhou, M. A survey of graph neural networks and their industrial applications. *Neurocomputing* 128761 (2024).

[20] Wang, D., Wei, W., Li, L., Wang, X. & Liang, J. Rethinking exploration–exploitation trade-off in reinforcement learning via cognitive consistency. *Neural Netw.* **187**, 107342 (2025). 40090299 10.1016/j.neunet.2025.107342

[21] Mnih, V. et al. Human-level control through deep reinforcement learning. *Nature* **518**, 7540, 529–533 (2015). 25719670 10.1038/nature14236

[22] Toan, N. D. & Gon-Woo, K. Environment exploration for mapless navigation based on deep reinforcement learning. In *21st International Conference on Control, Automation and Systems (ICCAS)*. 17–20 (2021).

[23] Auer, P. Using confidence bounds for exploitation-exploration trade-offs. *J. Mach. Learn. Res.* **3**, 397–422 (2003).

[24] Thompson, W. R. On the likelihood that one unknown probability exceeds another in view of the evidence of two samples. *Biometrika* **25**, 3–4 (1933).

[25] Kempka, M. et al. A doom-based AI research platform for visual reinforcement learning. In *IEEE Conference on Computational Intelligence and Games (CIG)*. 1–8. (2016).

[26] Chen, C. et al. Nuclear norm maximization-based curiosity-driven reinforcement learning. *IEEE Trans. Artif. Intell.* **5** (5), 2410–2421 (2024).

[27] Junjie, Z. et al. Exploration approaches in deep reinforcement learning based on intrinsic motivation: A review. *J. Comput. Res. Dev.* **60**, 2359–2382 (2023).

[28] Still, S. & Precup, D. An information-theoretic approach to curiosity-driven reinforcement learning. *Theory Biosci.* **131**, 139–148 (2012). 22791268 10.1007/s12064-011-0142-z

[29] Aubret, A., Matignon, L. & Hassas, S. An information-theoretic perspective on intrinsic motivation in reinforcement learning: A survey. *Entropy* **25** (2), 327 (2023). 36832693 10.3390/e25020327 PMC9954873

[30] Bellemare, M. G., Naddaf, Y., Veness, J. & Bowling, M. The arcade learning environment: an evaluation platform for general agents. *J. Artif. Intell. Res.* **47**, 253–279 (2013).

[31] Pang, G., van den Hengel, A., Shen, C. & Cao, L. Towards deep supervised anomaly detection: Reinforcement learning from partially labeled anomaly data. In *Proceedings of the 27th ACM SIGKDD Conference on Knowledge Discovery & Data Mining*. 1298–1308 (2021).

[32] Yang, K., Tao, J., Lyu, J. & Li, X. Exploration and anti-exploration with distributional random network distillation. In *Proceedings of the 41st International Conference on Machine LearningJMLR.org* (2024).

[33] Hu, Y. et al. Learning to utilize shaping rewards: A new approach of reward shaping. In *Proceedings of the 34th International Conference on Neural Information Processing Systems* (Curran Associates Inc., 2020).

[34] Almasan, P. et al. Deep reinforcement learning Meets graph neural networks: Exploring a routing optimization use case. *Comput. Commun.* **196**, 184–194 (2022).

[35] Wang, Z., Zhan, W., Duan, H. & Huang, H. Multiobjective optimization deep reinforcement learning for dependent task scheduling based on spatio-temporal fusion graph neural network. *Eng. Appl. Artif. Intell.* **148**, 110337 (2025).

[36] Ramesh, S., Sathyavarapu, N. S. B., Sharma, S. J. & Khanna, V. A. A. N. K. Comparative analysis of Q-learning, SARSA, and deep Q-network for microgrid energy management. *Sci. Rep.* **15**, 694 (2025). 39753622 10.1038/s41598-024-83625-8 PMC11698738

[37] Liu, G. et al. Supervised contrastive deep Q-network for imbalanced radar automatic target recognition. *Pattern Recogn.* **161**, 111264 (2025).

